# Rapid and motion-robust pediatric brain imaging: T2-weighted turbo-spin-echo PROPELLER acquisition with compressed sensing

**DOI:** 10.1007/s00247-024-06088-z

**Published:** 2024-11-26

**Authors:** Barbara Daria Wichtmann, Christoph Katemann, Mergim Kadrija, Yannik C. Layer, Leon M. Bischoff, Yvonne Scheuver, Madeleine Mezger, Oliver M. Weber, Julian A. Luetkens, Ulrike I. Attenberger, Alexander Radbruch, Daniel Paech

**Affiliations:** 1 Clinic of Neuroradiology, University Hospital Bonn, Venusberg-Campus 1, 53127 Bonn, Germany; 2 Department of Diagnostic and Interventional Radiology, University Hospital Bonn, Bonn, Germany; 3 Philips GmbH Market DACH, Hamburg, Germany; 4 Department of Radiology, Brigham and Women’s Hospital, Harvard Medical School, Boston, USA; 5 Department of Biomedical Imaging and Image-guided Therapy, Medical University of Vienna, Vienna, Austria

**Keywords:** Acceleration, Artifacts, Compressed sensing, Magnetic resonance imaging, Motion, Neuroimaging, Pediatric, PROPELLER

## Abstract

**Background:**

In pediatric magnetic resonance imaging (MRI), reducing the rate of non-diagnostic scans due to artifacts and shortening acquisition time are crucial not only for economic reasons but also to minimize sedation or general anesthesia.

**Objective:**

Enabling faster and motion-robust MRI of the brain in infants and children using a novel, enhanced compressed sensing (CS) algorithm in combination with a turbo-spin-echo T2-weighted sequence utilizing the PROPELLER-technique (**p**eriodically **r**otated **o**verlapping **p**arall**el** **l**ines with **e**nhanced **r**econstruction; T2_PROPELLER CS_).

**Materials and methods:**

This prospective study included 31 patients (8.0 ± 4.7 years, 15 males) undergoing a clinically indicated MRI examination of the brain on a 3-T scanner. The T2_PROPELLER CS_ sequence was compared to a conventional, CS-accelerated Cartesian turbo-spin-echo T2-weighted sequence (T2_Cartesian CS_). Apparent contrast-to-noise ratio (aCNR) and signal-to-noise ratio (aSNR) were calculated. Three blinded radiologists independently rated both sequences twice qualitatively on a 5-point Likert-scale from 1–5 (non-diagnostic–excellent) for artifacts, image sharpness, basal ganglia delineation, lesion conspicuity, and overall image quality. Statistical analysis was performed using the Wilcoxon signed-rank test and paired sample *t* test. Intra- and interrater reliability of qualitative image assessment was evaluated by computing Krippendorff’s $\alpha$ reliability estimates.

**Results:**

The average acquisition time of the T2_PROPELLER CS_ (189 ± 27 s) was 31% shorter than that of the T2_Cartesian CS_ sequence (273 ± 21 s; *P* < 0.001). aCNR (7.7 ± 4.6 vs. 6.2 ± 2.8; *P* = 0.004) and aSNR (24.8 ± 9.7 vs. 18.8 ± 5.5; *P* < 0.001) were higher for the T2_Cartesian CS_ compared to the T2_PROPELLER CS_ sequence. The T2_PROPELLER CS_ sequence significantly reduced (motion-)artifacts (*P* < 0.001) and increased image sharpness (*P* < 0.001), basal ganglia delineation (*P*<0.001), lesion conspicuity (raters 1 and 2, *P* < 0.001; rater 3, *P* = 0.004), and overall image quality (*P* < 0.001). Metal artifacts were prominent in both sequences, though slightly more pronounced in the T2_PROPELLER CS_ sequence.

**Conclusion:**

The T2_PROPELLER CS_ sequence enables faster and motion-robust imaging of the brain in infants and children, reducing the rate of non-diagnostic scans and potentially allowing sedation or general anesthesia to be minimized in the future.

**Graphical abstract:**

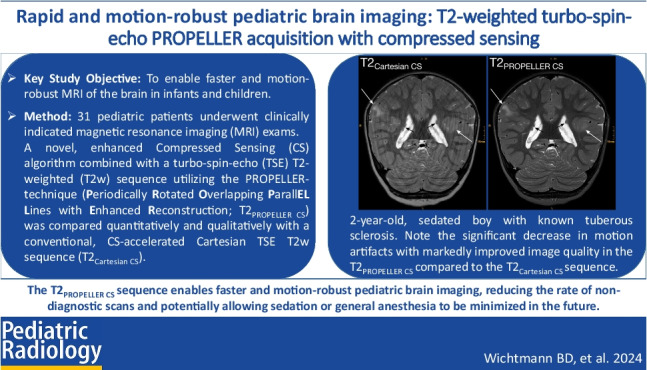

## Introduction

Owing to its exquisite soft tissue contrast and lack of ionizing radiation, magnetic resonance imaging (MRI) has become an indispensable diagnostic tool for brain imaging, especially in infants and children [1, 2]. However, the inherently long examination times of MRI are often challenging, as particularly young patients might not be able to cooperate by voluntarily constraining their movements causing artifacts and often requiring prolonged sedation or general anesthesia [3, 4]. Additionally, blood flow, heart, and respiratory rates are considerably higher in infants and children leading to an increased level of physiological noise [5]. To minimize the duration of sedation or general anesthesia and for economic reasons, the acquisition time as well as the rate of non-diagnostic scans should be reduced as much as possible [6, 7].

Various clinical accelerated MRI techniques have been developed over the last decades to alleviate the aforementioned limitations [8–13]. Compressed sensing (CS) is one such technique that exploits the implicit sparsity of MR images to significantly undersample k-space, thereby substantially speeding up imaging [14–17]. CS requires pseudo-random undersampling of k-space, which results in incoherent, i.e., noise-like, aliasing artifacts, which are subsequently reduced with an appropriate non-linear recovery scheme [14]. Clinically, a Cartesian sampling scheme is most commonly employed for its robust data collection, practical implementation, and simple reconstruction, except in cases where high temporal or spatial resolution is required [18]. However, Cartesian acquisition techniques are susceptible to motion artifacts because noise, either physiological or due to patient movements, propagates along a single phase-encoding direction as discrete ghost images. To mitigate artifacts arising from in-plane rotation and translation, non-Cartesian sampling schemes, such as radial trajectories, have been proposed that oversample the center of k-space, e.g., by rotating blades (**p**eriodically **r**otated **o**verlapping **p**arall**el** **l**ines with **e**nhanced **r**econstruction (PROPELLER); [3, 19–22]). Variants of this method have been implemented by various vendors under different proprietary names: BLADE (Siemens Healthcare, Erlangen, Germany), PROPELLER (GE Medical Systems, Milwaukee, Wis), and MultiVane (Philips, Best, the Netherlands; [23]). Yet, a clinically implementable CS solution for the PROPELLER approach has only very recently been introduced [24, 25].

In this study, we evaluated a novel, clinically feasible, enhanced CS algorithm combined with a turbo-spin-echo (TSE) T2-weighted (T2w) sequence using the PROPELLER-technique (SmartSpeed MotionFree; Philips Healthcare, Best, the Netherlands) that has been shown to provide improved motion correction and contrast weighting over the conventional T2w PROPELLER sequence [24]. The study’s objective was to achieve faster and motion-robust MR imaging of the brain in infants and children. We compared this novel sequence with the conventional, CS-accelerated Cartesian TSE T2w sequence qualitatively in terms of artifacts, image sharpness, basal ganglia delineation, lesion conspicuity, and overall image quality, as well as quantitatively in terms of the apparent contrast-to-noise ratio (aCNR), apparent signal-to-noise ratio (aSNR), and acquisition time. Particularly in pediatric brain imaging, reducing acquisition time while maintaining or even improving image quality is critical to ensure safe, rapid, and robust examinations of these young patients.

## Materials and methods

### Study population

This institutional review board-approved prospective study enrolled 31 patients (mean ± standard deviation (SD) age, 8.0 ± 4.7 years; age range, 0.6–16.5 years; 15 males), who were referred to the radiology department for a clinically indicated MRI examination of the brain. Indications included congenital or acquired hydrocephalus after intraventricular hemorrhage in prematurity; neoplastic conditions or other mass or mass-like conditions and oncological follow-up; epilepsy and movement disorders; evaluation of headaches with suspected brain structural abnormality; congenital disorders and anatomical abnormalities; pediatric neurodegenerative disorders; vascular, i.e., arteriovenous malformation; neurometabolic disorder, i.e., pyruvate dehydrogenase deficiency; trauma including diffuse axonal injury; and elevated intracranial pressure.

All patients’ legal guardians gave written informed consent prior to participation in the study. Participants were consecutively recruited from March to June 2022 and in November 2023. Eighteen patients were sedated (mean ± SD age, 5.0 ± 2.9 years; age range, 0.6–13.3 years), and 13 patients were awake, i.e., not sedated, for their MRI examination (mean ± SD age, 12.3 ± 3.3 years; age range, 6.0–16.5 years).

### Data acquisition

All brain scans were performed on a clinical 3-T MRI scanner (Ingenia 3.0 T ElitionX, Philips Healthcare, Best, the Netherlands) using the standard head coil. MRI protocols were set up to answer the respective clinical questions per patient, typically comprising the following sequences: transverse T2w fluid-attenuated inversion recovery (FLAIR), transverse diffusion-weighted imaging (DWI), transverse susceptibility-weighted MRI (SWI), either transverse or in three specified instances coronal CS-accelerated Cartesian TSE T2w, and a sagittal 3D T1-weighted (T1w) turbo field echo (TFE) without contrast enhancement and in selected cases additionally with contrast enhancement. For all patients, the CS-accelerated TSE T2w sequence using the PROPELLER-technique (henceforth named “T2_PROPELLER CS_”) was acquired in the same orientation as the conventional CS-accelerated Cartesian TSE T2w sequence (henceforth named “T2_Cartesian CS_”). The T2_PROPELLER CS_ sequence was acquired before the T2_Cartesian CS_ sequence for 4 of the anesthetized patients and after for the remaining patients.

In both scans, the employed CS technique was based on a combination of compressed sensing and parallel imaging using SENSE (Compressed SENSE, Philips Healthcare, Best, The Netherlands). Imaging parameters per sequence are provided in Table 1.

**Table 1:** Imaging parameters

	Transverse T2_Cartesian CS_	Transverse T2_PROPELLER CS_	Coronal T2_Cartesian CS_	Coronal T2_PROPELLER CS_
Echo time (TE) [ms]	90	100	90	100/110
Repetition time (TR) [ms]	6,316–7,369	6,107–7,968	3,981–6,637	5,393–7,968
Flip angle [degree °]	90	90	90	90
Acquired voxel size [mm^3^]	0.55 × 0.55 × 2	0.55 × 0.55 × 2	0.55 × 0.55 × 2	0.55 × 0.55 × 2
Reconstructed voxel size [mm^3^]	0.18 × 0.18 × 2	0.41 × 0.41 × 2	0.18 × 0.18 × 2	0.41 × 0.41 × 2
Bandwidth [Hz/pixel]	217/219	265	217	265/268
CS-factor	2/2.5	2	2	2/2.5
Acquisition time (mean ± SD) [s]	277 ± 8	192 ± 28	256 ± 44	176 ± 19

This table lists parameters of the conventional compressed sensing (*CS*) accelerated Cartesian turbo-spin-echo (*TSE*) T2-weighted (*T2w*) sequence (T2_Cartesian CS_) against an enhanced CS algorithm in combination with a TSE T2w sequence using the *PROPELLER*-technique (**p**eriodically **r**otated **o**verlapping **p**arall**el** **l**ines with **e**nhanced **r**econstruction; T2_PROPELLER CS_). SI/non-SI accepted units of the respective parameters are provided in square brackets

*CS* compressed sensing, *PROPELLER* **p**eriodically **r**otated **o**verlapping **p**arall**el** **l**ines with **e**nhanced **r**econstruction, *SD* standard deviation, *SI* Système international d'unités, *T2w* T2-weighted, *TSE* turbo-spin-echo

### Image analysis

**Fig. 1:**
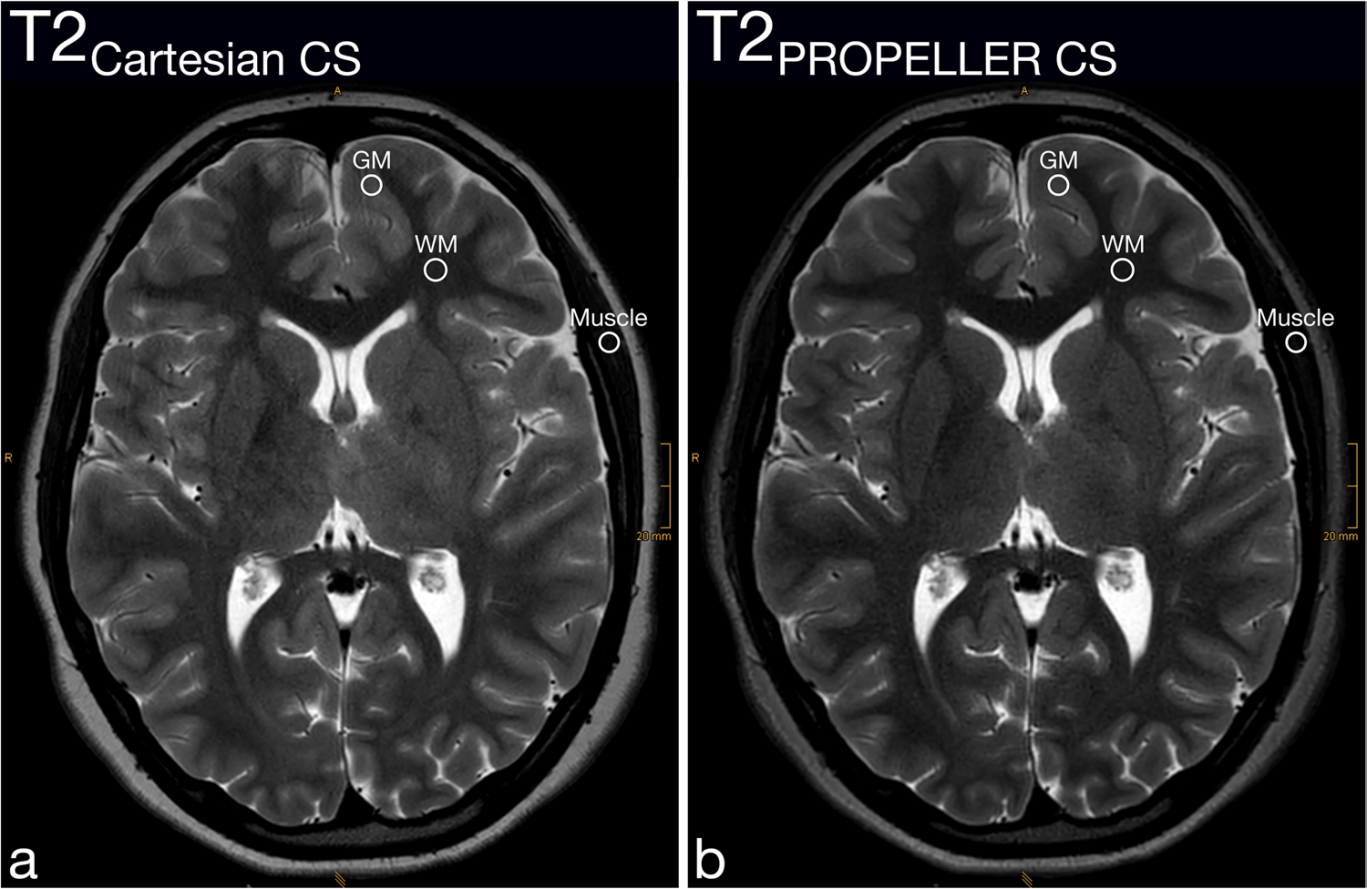
Shown are axial T2_Cartesian CS_ (**a**) and T2_PROPELLER CS_ (**b**) images of a 15-year-old, non-sedated girl with a low-grade astrocytoma extending into the basal ganglia and thalamus on the left. For the quantitative image analysis, regions of interest (ROIs) were consistently placed in the frontal cortex (gray matter, *GM*), frontal white matter (*WM*), and musculus temporalis (*Muscle*) in identical positions in both sequences

Based on the image intensity distribution, i.e., mean and SD of defined regions of interest (ROIs), the aCNR of gray/white matter (GM/WM) (aCNR = (mean(GM)-mean(WM))/SD(muscle)) and aSNR of GM (aSNR = mean(GM)/SD(muscle)) were calculated for all images [24, 26–29]. ROIs were consistently placed in the frontal cortex (GM), frontal WM, and temporalis muscle (muscle) in identical positions in both sequences, as depicted in Fig. 1.

Three blinded radiologists with 10 years (rater 1), 6 years (rater 2), and 3 years (rater 3) of experience independently rated both sequences, T2_Cartesian CS_ and T2_PROPELLER CS_, qualitatively on a 5-point Likert-scale from 1–5 (non-diagnostic–excellent) for artifacts, image sharpness, basal ganglia delineation, lesion conspicuity, and overall image quality. Five-point Likert items were defined with respect to the different categories as listed in Table 2. To assess intrarater reliability, all raters repeated the rating for a second time at least 2 weeks after the initial rating.

**Table 2:** Five-point Likert-scale per category

5-point Likert item	Artifacts	Image sharpness Basal ganglia delineation Lesion conspicuity Overall image quality
1	Non-diagnostic	Non-diagnostic, blurry
2	Severe artifacts with insufficient diagnostic confidence	Poor, structures can be defined but insufficient diagnostic confidence
3	Moderate artifacts, not inferring with diagnosis	Moderate, sufficient for diagnosis but low diagnostic confidence
4	Minimal artifacts	Good, diagnostic with high diagnostic confidence
5	No artifacts	Excellent, sharp images with exceptional diagnostic confidence

Three blinded radiologists independently rated both sequences, T2_Cartesian CS_ and T2_PROPELLER CS_, qualitatively on a 5-point Likert-scale from 1–5 (non-diagnostic–excellent) for artifacts, image sharpness, basal ganglia delineation, lesion conspicuity, and overall image quality. This table lists the definition of the 5-point Likert items with respect to the different categories

### Statistical analysis

Statistical analysis and visualization were performed in MATLAB (The MathWorks Inc. (2020), MATLAB Version: 9.9.0 (R2020b)) using the Wilcoxon signed-rank test and paired sample *t* test. A *P*-value of < 0.05 was considered significant.

Intra- and interrater reliability of qualitative image assessment was evaluated by computing Krippendorff’s $\alpha$ reliability estimates using the freely available macro written for SPSS (IBM SPSS Statistics version 27) taking 10,000 bootstrap samples to determine their corresponding 95% confidence intervals (CIs) [30, 31]. Krippendorff’s $\alpha$ can handle various numbers of raters and applies to any measurement level, including the ordinal Likert-scale. Krippendorff’s *α* = 0 indicates no and *α* = 1 perfect reliability, whereas values < 0 are achieved when disagreements are systematic and exceed what can be expected by chance [32].

In the following, continuous variables of the quantitative assessment are provided as mean ± SD and discrete variables of the qualitative assessment as median with interquartile range (IQR).

## Results

### Quantitative evaluation

The average acquisition time of the T2_PROPELLER CS_ and T2_Cartesian CS_ sequences in transverse and coronal orientation is provided in Table 1. Overall, the average acquisition time of the T2_PROPELLER CS_ sequence (189 ± 27 seconds (s)) was 31% shorter than that of the T2_Cartesian CS_ sequence (273 ± 21 s; *P* < 0.001).

Quantitative image analysis yielded higher aCNR (7.7 ± 4.6 vs. 6.2 ± 2.8; *P* = 0.004) and aSNR (24.8 ± 9.7 vs. 18.8 ± 5.5; *P* < 0.001) for the T2_Cartesian CS_ compared to the T2_PROPELLER CS_ sequence.

### Qualitative evaluation

Intrarater reliability for qualitative scoring was excellent with reliabilities of *α* = 0.92 (95% CI, 0.90–0.94; rater 1), *α* = 0.94 (95% CI, 0.91–0.96; rater 2), and *α* = 0.92 (95% CI, 0.88–0.95; rater 3). Interrater agreement was reliable with an $\alpha$ of 0.73 (95% CI, 0.70–0.76).

Table 3 provides the results of the qualitative rating of each individual rater at both time points, along with the *P*-values indicating the significance of the differences between the two sequences.

**Table 3:** Qualitative evaluation per rater

Rater	Rating	Artifacts			Image sharpness			Basal ganglia delineation			Lesion conspicuity			Overall image quality		
		T2_Cartesian CS_	T2_PROPELLER CS_	*P*	T2_Cartesian CS_	T2_PROPELLER CS_	*P*	T2_Cartesian CS_	T2_PROPELLER CS_	*P*	T2_Cartesian CS_	T2_PROPELLER CS_	*P*	T2_Cartesian CS_	T2_PROPELLER CS_	*P*
1	1	3 (2–3)	4 (4–5)	** < 0.001**	3 (3–3.75)	4 (4–5)	** < 0.001**	3 (3–3)	5 (4–5)	** < 0.001**	3 (2.75–3)	4 (4–4.25)	** < 0.001**	3 (3–3)	5 (4–5)	** < 0.001**
	2	3 (2–3)	4 (4–5)	** < 0.001**	3 (3–3)	4 (4–5)	** < 0.001**	3 (3–3)	5 (4–5)	** < 0.001**	3 (2–3)	4 (4–4.25)	** < 0.001**	3 (3–3)	5 (4–5)	** < 0.001**
2	1	3 (3–3.75)	4 (4–5)	** < 0.001**	3 (3–4)	5 (4–5)	** < 0.001**	3 (3–3)	5 (4–5)	** < 0.001**	3 (3–3.25)	4 (4–4)	** < 0.001**	3 (3–3.75)	4 (4–5)	** < 0.001**
	2	3 (2–4)	5 (4–5)	** < 0.001**	3 (3–4)	5 (4.25–5)	** < 0.001**	3 (2.25–3)	5 (4–5)	** < 0.001**	3 (3–3.25)	4 (4–4)	** < 0.001**	3 (3–3.75)	5 (4–5)	** < 0.001**
3	1	4 (3–4)	5 (4–5)	** < 0.001**	3 (3–4)	4 (4–5)	** < 0.001**	3 (3–4)	4 (4–5)	** < 0.001**	4 (3–4)	4 (4–4)	**0.004**	3 (3–4)	4 (4–5)	** < 0.001**
	2	3 (3–4)	5 (4.25–5)	** < 0.001**	3 (3–4)	4 (4–5)	** < 0.001**	3 (3–4)	4 (4–5)	** < 0.001**	4 (3–4)	4 (4–4)	**0.004**	3 (3–4)	4 (4–5)	** < 0.001**

Results of the qualitative evaluation of the T2_Cartesian CS_ and T2_PROPELLER CS_ sequence performed by three independent raters (rater 1–3) at two different time points (ratings 1 and 2), along with the *P*-values indicating the significance of the differences between the two sequences. Ratings are based on a 5-point Likert-scale from 1–5 (non-diagnostic–excellent) and provided as median with interquartile range (IQR) for each evaluated category, i.e., artifacts, images sharpness, basal ganglia delineation, lesion conspicuity, and overall image quality. Values in bold indicate statistically significant differences. All raters rated the T2_PROPELLER CS_ sequence significantly higher than the T2_Cartesian CS_ sequence across all assessed categories

**Fig. 2:**
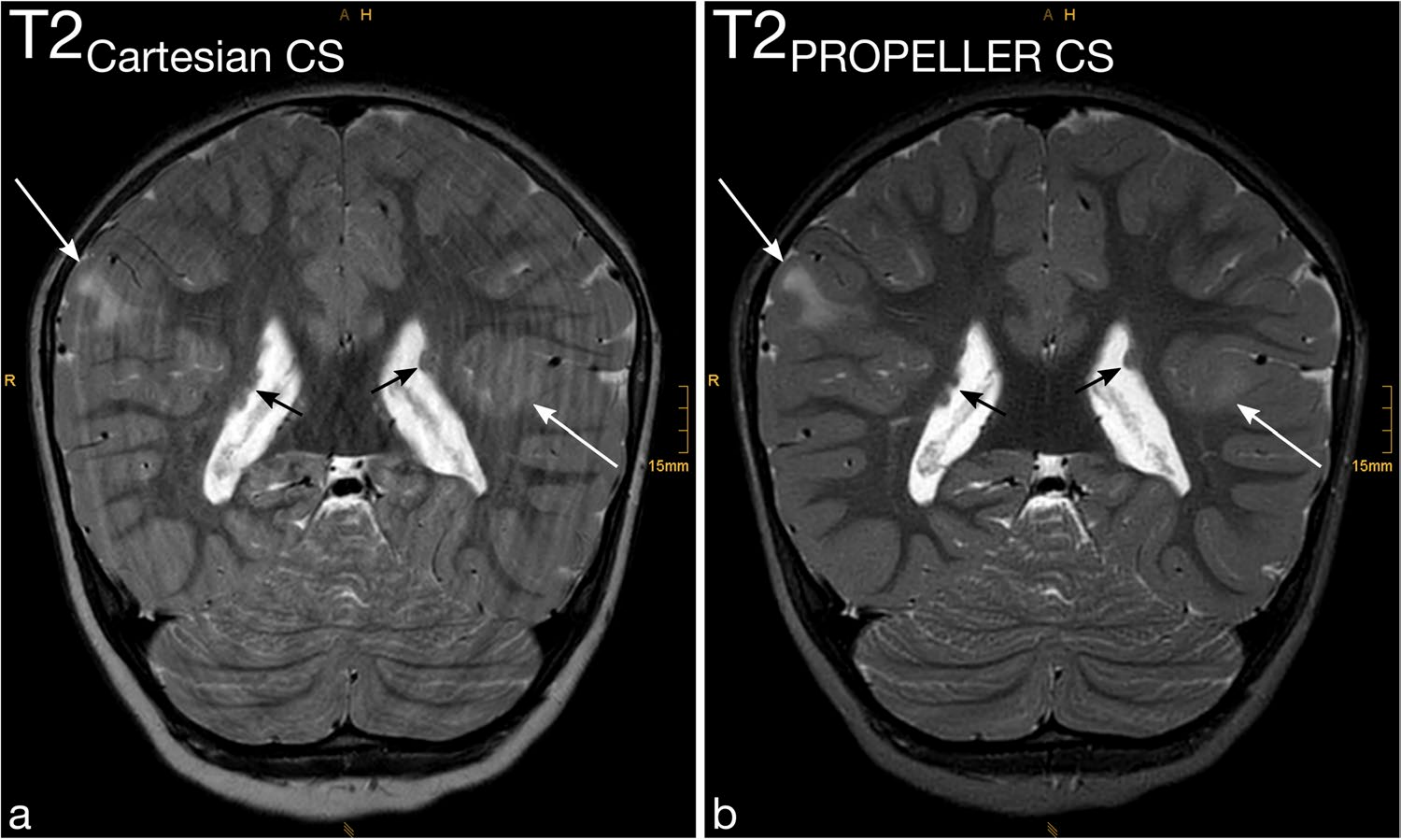
Coronal T2_Cartesian CS_ (**a**) and T2_PROPELLER CS_ (**b**) images of a 2-year-old, sedated boy with known tuberous sclerosis, developmental delay, and West syndrome. Due to the extensive motion artifacts, the cortical/subcortical tuberosities (*white long arrows*) and the subependymal hamartomas (*black short arrows*) are poorly or indistinctly delineated in the conventional T2_Cartesian CS_ sequence (**a**). Note the significant decrease in motion artifacts with markedly improved image quality in the T2_PROPELLER CS_ sequence (**b**)

**Fig. 3:**
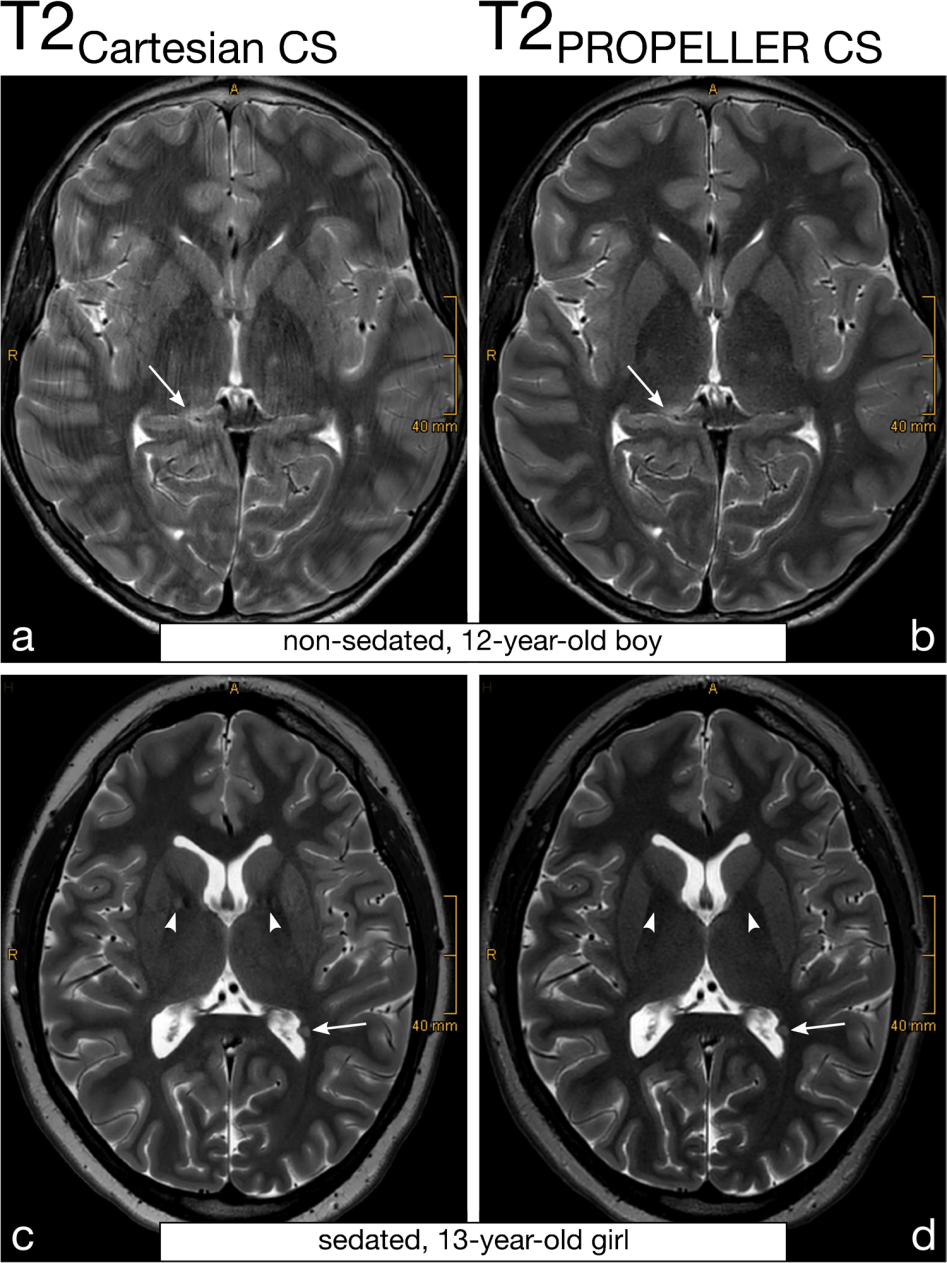
Comparison of the performance of the axial T2_PROPELLER CS_ sequence (**b** and **d**) against the T2_Cartesian CS_ sequence (**a** and **c**) in a non-sedated, 12-year-and-8-month-old boy with epilepsy (**a** and **b**) and a sedated, 13-year-and-3-month-old girl with a neurodegenerative disorder (**c** and **d**). Non-sedated patient (**a** and **b**): The T2_Cartesian CS_ sequence (**a**) is prone to motion artifacts, resulting in an image of poor diagnostic quality. Note the low conspicuity of the T2-hyperintense lesion in the right thalamus in the T2_Cartesian CS_ sequence (**a**, *arrow*), whereas the T2_PROPELLER CS_ sequence (**b**) exhibits no motion corruption with excellent lesion conspicuity (**b**, *arrow*). Sedated patient (**c** and **d**): The T2_PROPELLER CS_ sequence (**d**) notably demonstrates superior suppression of physiological noise, i.e., the pulsation artifacts present in the T2_Cartesian CS_ sequence (**c**, *arrowheads*) are not observed in the T2_PROPELLER CS_ sequence (**d**, *arrowheads*). The nodular heterotopia at the posterior horn of the left lateral ventricle is comparably well delineated in both sequences (**c** and **d**, *arrows*)

**Fig. 4:**
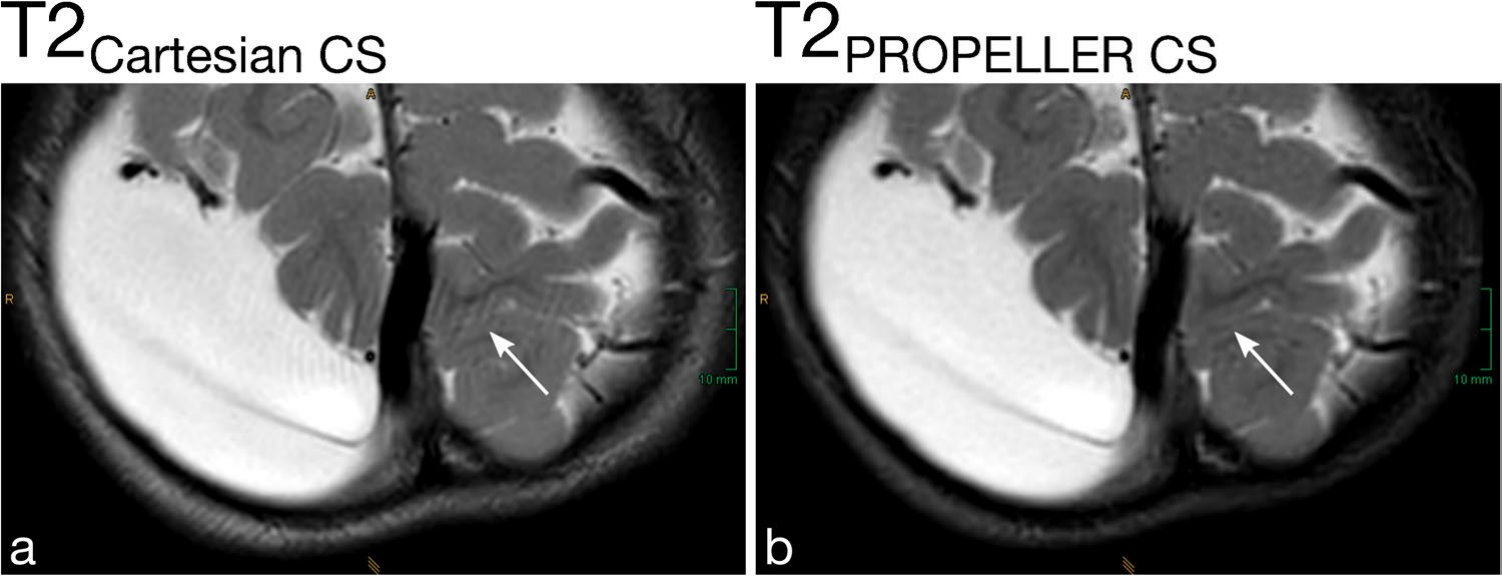
Detailed views of the axial T2_Cartesian CS_ (**a**) and T2_PROPELLER CS_ (**b**) sequences posteriorly at the level of the superior sagittal sinus of a 3-year-old, sedated girl post surgery for post-hemorrhagic hydrocephalus after intraventricular hemorrhage grade III in prematurity. Note the reduction of pulsation and Gibbs artifacts in the T2_PROPELLER CS_ (**b**) compared to the T2_Cartesian CS_ (**a**) sequence (*arrows*)

**Fig. 5:**
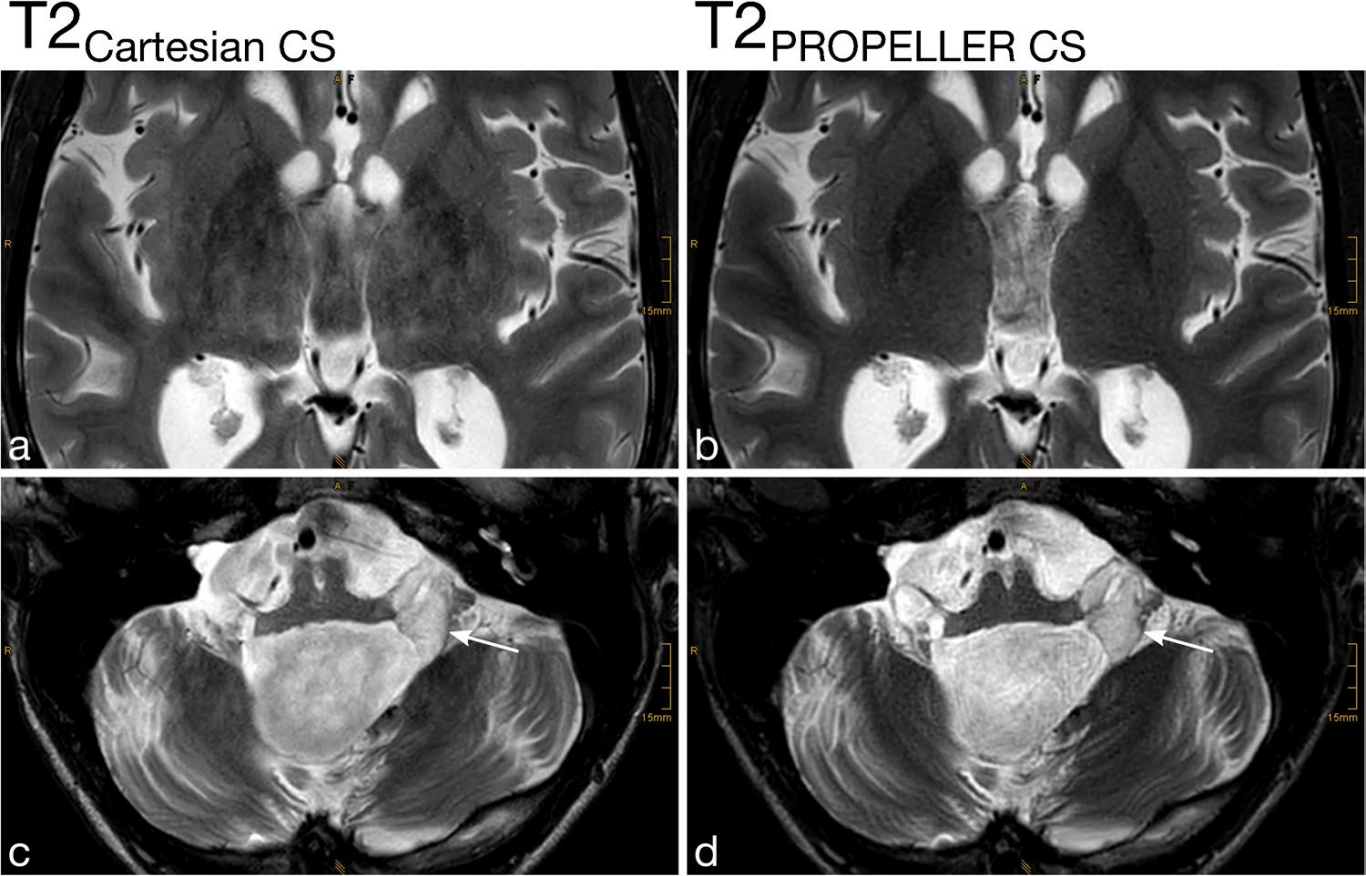
Detailed views of the axial T2_Cartesian CS_ (**a** and **c**) and T2_PROPELLER CS_ (**b** and **d**) sequences at the level of the basal ganglia (**a** and **b**) and cerebellopontine angle (**c** and **d**) of a 15-year-old, non-sedated boy after resection of a pilocytic astrocytoma infratentorially with residual tumor components in the left cerebellopontine angle (**c** and **d**, *white arrows*). The markedly increased physiological noise and pulsation artifacts in the T2_Cartesian CS_ sequence (**a** and **c**) result in poor delineation of both the basal ganglia and the lesion. The T2_PROPELLER CS_ sequence (**b** and **d**) reduces artifacts and increases image sharpness, basal ganglia delineation, lesion conspicuity, and overall image quality

**Fig. 6:**
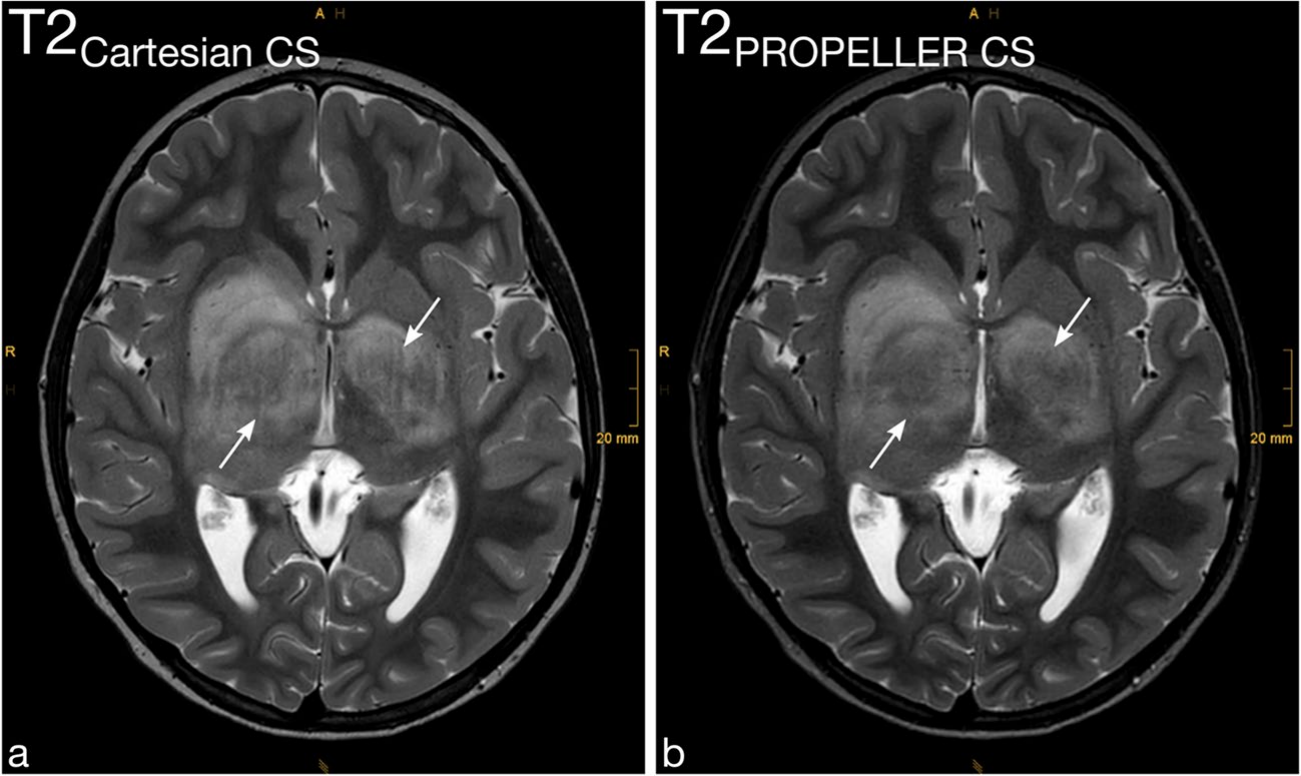
Axial T2_Cartesian CS_ (**a**) and T2_PROPELLER CS_ (**b**) images of a 6-year-old, sedated girl with central nervous system manifestations of neurofibromatosis type 1 with bilateral gliomas of the basal ganglia. Note the increased level of physiological noise in the T2_Cartesian CS_ sequence (**a**) with pulsation artifacts running through the lesions (*arrows*) compared to the T2_PROPELLER CS_ sequence (**b**) with improved image quality

**Fig. 7:**
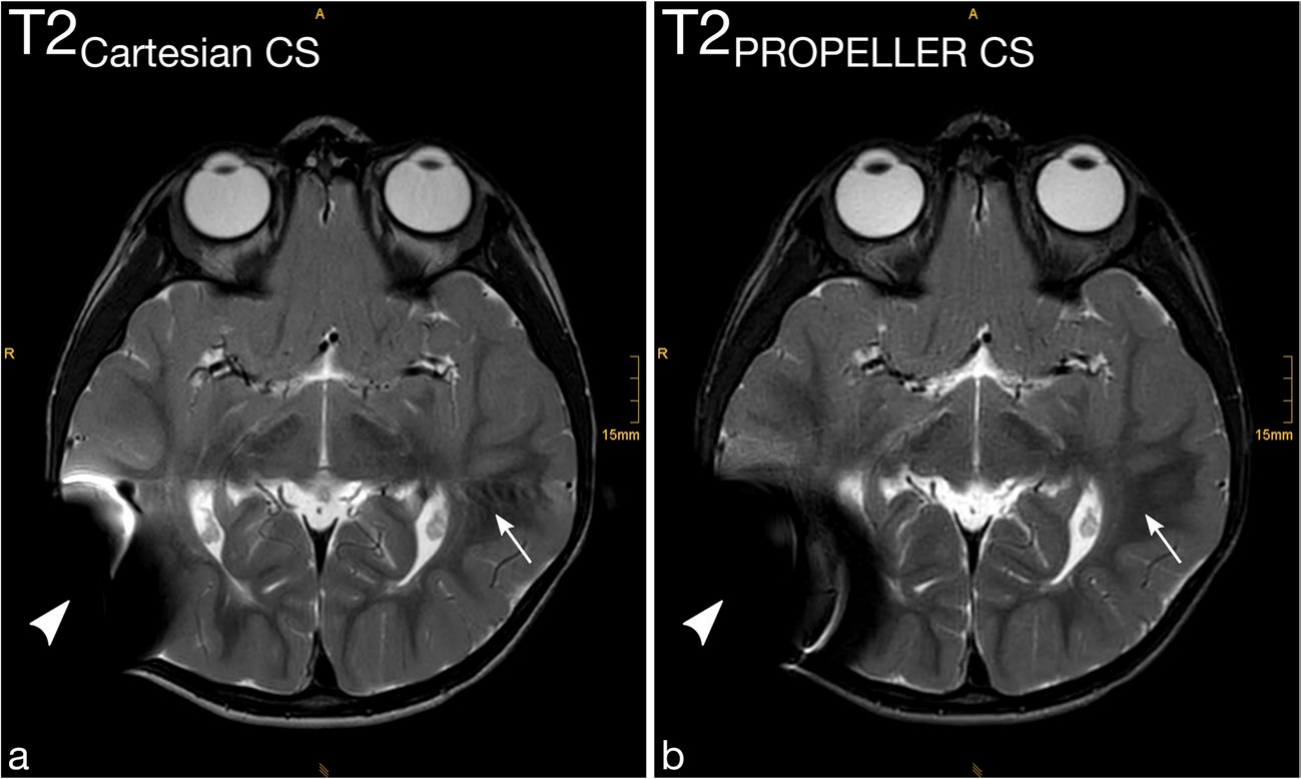
Axial T2_Cartesian CS_ (**a**) and T2_PROPELLER CS_ (**b**) images of a 3-year-old, sedated girl, former preterm infant (28th week of gestation) with positional plagiocephalus and placement of a left frontal Rickham reservoir for post-hemorrhagic hydrocephalus. Note the locally accentuated metal artifacts of the right parietal shunt valve in both sequences, which are slightly more pronounced in the T2_PROPELLER CS_ sequence (*arrowheads*). In the T2_Cartesian CS_ sequence, we observe an additional Zipper artifact in phase-encoding direction at the level of the shunt valve that is not present in the T2_PROPELLER CS_ sequence (*arrows*)

Motion artifacts (Fig. 2 and Fig. 3), Gibbs artifacts, and physiological noise, including CSF- and blood-flow artifacts (Fig. 3, Fig. 4, Fig. 5, and Fig. 6), were significantly reduced in the T2_PROPELLER CS_ compared to the T2_Cartesian CS_ sequence. Particularly in patients without sedation, motion artifacts were notably pronounced in the T2_Cartesian CS_ sequence resulting at times in non-diagnostic images, while the afterwards acquired T2_PROPELLER CS_ sequence yielded images with good to excellent quality (Fig. 3). In 5 patients, a shunt valve caused prominent metal artifacts in both sequences, which were slightly more pronounced locally in the T2_PROPELLER CS_ sequence. In the T2_Cartesian CS_ sequence, we observed an additional Zipper artifact in phase-encoding direction at the level of the shunt valve (Fig. 7).

Image sharpness was rated significantly higher for the T2_PROPELLER CS_. Alongside, basal ganglia delineation was significantly increased in the T2_PROPELLER CS_ compared to the T2_Cartesian CS_ sequence (Fig. 5 and Fig. 6).

Twenty-one of the 31 patients had lesions, including pre- and post-treatment astrocytic tumors such as pilocytic astrocytomas of the cerebellum (Fig. 5) or low-grade optic pathway and thalamus gliomas (Fig. 1) including central nervous system manifestations of neurofibromatosis type 1 (Fig. 6); diffuse midline gliomas; post-treatment changes, e.g., at the cerebellar resection margin of a medulloblastoma and after resection of Langerhans cell histiocytosis of the left mastoid; suspected dysembryoplastic neuroepithelial tumor in the left parahippocampal gyrus; cystic lesions such as a pineal cyst, a cystic defect in the left parathalamic region or an atypical Virchow-Robin space; tuberous sclerosis with subcortical/cortical tubers and subependymal hamartomas (Fig. 2); nodular heterotopias in the lateral ventricles (Fig. 3); suspected post-inflammatory changes in the right thalamus (Fig. 3); nodular protrusion in the right pulvinar nuclei; ventriculoperitoneal shunt placement for post-hemorrhagic hydrocephalus; and axonal shear trauma with multiple post-hemorrhages defects. Overall, lesions were significantly more conspicuous in the T2_PROPELLER CS_ compared to the T2_Cartesian CS_ sequence.

Both raters evaluated the overall image quality of the T2_PROPELLER CS_ sequence significantly higher than of the T2_Cartesian CS_ sequence (Fig. 1, Fig. 2, Fig. 3, Fig. 4, Fig. 5, and Fig. 6).

Irrespective of the acquisition order, the T2_PROPELLER CS_ sequence was rated in all categories equal or superior to the T2_Cartesian CS_ sequence.

**Fig. 8:**
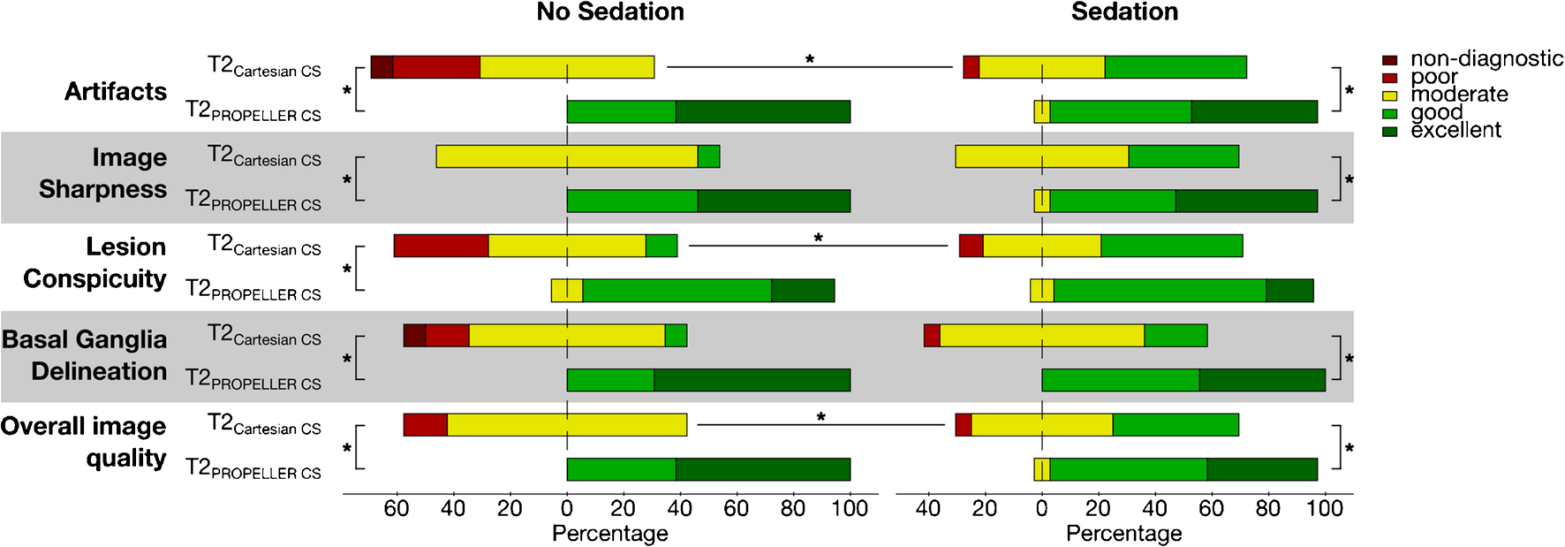
Results of the first qualitative assessment of the T2_Cartesian CS_ and T2_PROPELLER CS_ sequences performed independently by three blinded radiologists. Median ratings are compared for non-sedated and sedated patients. Statistically significant differences are indicated by an *asterisk* (*). The applied 5-point Likert-scale ranged from 1 (dark red, non-diagnostic) to 5 (dark green, excellent); for the exact definition, see Table 2. Irrespective of sedation, the T2_PROPELLER CS_ sequence was rated superior to the T2_Cartesian CS_ sequence in all evaluated categories, i.e., artifacts, image sharpness, lesion conspicuity, basal ganglia delineation, and overall image quality. The T2_Cartesian CS_ sequence exhibited significantly more pronounced artifacts in the non-sedated patient group compared to the sedated patient group, resulting in poor and sometimes non-diagnostic scans. In contrast, the T2_PROPELLER CS_ sequence yielded no significant differences between the sedated and non-sedated patient collective with images generally rated as good to excellent

Figure 8 compares the median ratings of the two sequences across the different categories for sedated versus non-sedated patients. Apart from its aforementioned superiority in all categories, the T2_PROPELLER CS_ sequence demonstrated no significant differences between the sedated and non-sedated patient collective, with images generally rated as of good to excellent quality. In contrast, the T2_Cartesian CS_ sequence exhibited significantly more pronounced artifacts and decreased lesion conspicuity in the non-sedated patient group, resulting in poor and sometimes non-diagnostic scans.

## Discussion

In this study, we evaluated a new, clinically feasible, enhanced CS algorithm combined with a TSE T2w sequence using the PROPELLER-technique (T2_PROPELLER CS_) against a conventional, CS-accelerated Cartesian TSE T2w sequence (T2_Cartesian CS_) for MR imaging of the brain in infants and children. In comparison to the conventional T2_Cartesian CS_ sequence, the T2_PROPELLER CS_ sequence enabled faster and motion-robust imaging significantly accelerating acquisition time by 31%, reducing (motion-)artifacts, increasing image sharpness, basal ganglia delineation, lesion conspicuity, and overall image quality. aCNR and aSNR were reduced without significantly affecting the diagnostic value.

In pediatric MRI, reducing the rate of non-diagnostic scans due to artifacts and shortening acquisition time are crucial not only for economic reasons but also to minimize sedation or general anesthesia. Despite strategies such as exam preparation with MRI simulation and in-scan entertainment, the MRI environment is often intimidating and requires sedation or general anesthesia for children under the age of 6 or those with serious illnesses and difficulties to remain still during lengthy exams [33]. While sedation is generally well tolerated by children, the potential risks of increased doses of anesthetic medications during prolonged MRI examinations and the economic as well as operational expenses associated with the use of anesthesia underscore the need to minimize the use of anesthesia and decrease scan durations [33].

Over the last decade, sparse reconstruction techniques, including the CS framework, have been extensively investigated for their potential to decrease scan durations in neuroimaging and pediatric MRI [8–10]. The need for speed concerns not only the scan duration itself, but also the robustness and the efficiency of the data acquisition [11]. Besides minimizing the effects of involuntary motion and physiological noise by reducing the acquisition time, motion artifacts due to in-plane rotation and translation can be further avoided by combining CS with radial sampling trajectories such as the PROPELLER-technique that oversample the center of k-space [34, 35]. Yet only recently a CS solution for the PROPELLER approach has become clinically available which allows a significant reduction in acquisition time [24, 25].

During the re-gridding process of the PROPELLER-technique, any k-space data that was compromised by motion is dispersed through the Cartesian-like matrix, reducing the impact of motion [3]. Even though the T2_PROPELLER CS_ sequence in this study was acquired in non-sedated patients after the T2_Cartesian CS_ sequence toward the end of the MRI exam when children tended to be more agitated, it provided images without impairment by motion artifacts that were of good to excellent quality and rated superior to the conventional images by three experienced radiologists in all evaluated categories [36]. Particularly, no significant differences in image quality were observed between sedated and non-sedated patients for the T2_PROPELLER CS_ sequence underscoring its effectiveness in reducing artifacts. In contrast, the conventional T2_Cartesian CS_ sequence was highly susceptible to motion artifacts that were significantly more pronounced in patients without sedation, resulting in images of non-diagnostic or poor quality that required repeat acquisition. Beyond motion artifacts, physiological noise was also significantly reduced in the T2_PROPELLER CS_ sequence irrespective of sedation. The diagnostic superiority of the new sequence is further supported by the significantly improved lesion conspicuity, basal ganglia delineation, image sharpness, and overall image quality, regardless of whether the children were sedated or not. Analogous to our study, Vertinsky et al. were able to demonstrate the superior motion artifact reduction and diagnostic confidence of a T2w PROPELLER TSE against a conventional T2w TSE sequence and concluded that particularly young infants not undergoing anesthesia will benefit from the PROPELLER-technique [22]. Forbes et al. compared a PROPELLER T2w TSE sequence with enhanced reconstruction to a T2w single-shot TSE sequence in unsedated pediatric patients and found them to provide equal motion correction, with the PROPELLER approach enabling better assessment of the brain parenchyma [37]. Further accelerating the PROPELLER T2w TSE sequence using the new, clinically feasible, enhanced CS algorithm with improved motion correction and contrast weighting featured in this study holds the potential to further minimize or potentially even circumvent sedation time [6]. Moreover, shorter examinations allow for increased patient throughput, offering further financial incentives. Lastly, Andre et al. examined the prevalence, severity, and cost associated with motion artifacts and found that they are a common cause of MR image degradation and impose significant costs on radiology departments [38].

In this study, aCNR and aSNR of the T2_PROPELLER CS_ sequence were reduced in comparison to the T2_Cartesian CS_ sequence. While the PROPELLER-technique oversamples the k-space center, which typically improves the CNR and partially enhances the SNR, SNR still poses a significant challenge particularly when combined with acceleration techniques of any kind [3]. The increased bandwidth of the T2_PROPELLER CS_ sequence in comparison to the T2_Cartesian CS_ sequence may additionally contribute to the reduced aCNR and aSNR of the T2_PROPELLER CS_ sequence in this study. Still, it is to note that the decreased aCNR and aSNR of the T2_PROPELLER CS_ sequence did not affect the overall image quality as all radiologists preferred the T2_PROPELLER CS_ over the T2_Cartesian CS_ sequence. Metal artifacts were prominent in both sequences, though locally slightly more pronounced in the T2_PROPELLER CS_ sequence. In the T2_Cartesian CS_ sequence, we observed an additional Zipper artifact in phase-encoding direction at the level of the shunt valve that was not present in the T2_PROPELLER CS_ sequence. Due to the rotating frequency encoding direction, the radial acquisition scheme of the T2_PROPELLER CS_ sequence results in more pronounced local susceptibility artifacts. This is counteracted with a higher bandwidth, which in turn results in a slightly reduced aSNR. Zipper artifacts along the phase-encoding direction in the T2_Cartesian CS_ sequence caused by imperfections of the CS reconstruction due to strong B0 inhomogeneities are avoided by the rotating phase encoding in the T2_PROPELLER CS_ sequence.

### Limitations

Our study has several limitations. While we enrolled pediatric patients referred for brain MRI for a wide variety of indications, the relatively short study period of a few months and the inclusion of just more than 30 study patients may limit the generalizability of our findings. Future studies with larger patient populations will be valuable to further assess the performance of the T2_PROPELLER CS_ sequence in routine clinical practice.

Furthermore, although we demonstrated good intra- and interrater reliability, involving more reviewers with varying levels of experience could further enhance the consistency and objectivity of image interpretation.

We utilized a high-end, clinical 3-T MRI system with advanced reconstruction hardware, resulting in short reconstruction times of around 30 s for the CS images. Yet, it is important to consider that image quality and acquisition as well as reconstruction times may vary depending on the specific MRI system employed [11, 34]. In particular, applicability of the T2_PROPELLER CS_ sequence on a 1.5-T MRI system remains to be evaluated.

Lastly, this study is an initial evaluation of the T2_PROPELLER CS_ sequence. Future studies will focus on further optimization by pushing the undersampling limits to further accelerate imaging and enhance diagnostic efficiency.

## Conclusion

The T2_PROPELLER CS_ sequence enables faster and motion-robust imaging of the brain in infants and children, potentially reducing the rate of non-diagnostic scans and sedation or general anesthesia time.

## Data Availability

Participants of this study did not agree for their data to be shared publicly. Identifying facial information can be reconstructed from brain images. Therefore, supporting data are not available.
